# The impact of malocclusion on the oral health related quality of life of 11–14-year-old children

**DOI:** 10.1186/s12887-022-03127-2

**Published:** 2022-02-14

**Authors:** Jagan K. Baskaradoss, Amrita Geevarghese, Waad Alsaadi, Huda Alemam, Amjad Alghaihab, Amal Saad Almutairi, Abeer Almthen

**Affiliations:** 1 Faculty of Dentistry, Division of Dental Public Health, Department of Developmental and Preventive Sciences, Kuwait University, Safat-13110, P.O. Box: 24923, Kuwait City, Kuwait; 2 Division of Oral Epidemiology and Dental Public Health, Department of Preventive and Restorative Dental Sciences, School of Dentistry, University of California San Francisco, San Francisco, CA USA; 3 Department of Pediatric Dentistry and Orthodontics, College of Dentistry, King Saud University, Riyadh, Saudi Arabia; 4 Saudi board of pediatric dentistry, Riyadh, Saudi Arabia; 5 Department of Maxillofacial Surgery and Diagnostic Sciences, College of Dentistry, King Saud bin Abdulaziz University for Health Sciences, Riyadh, Saudi Arabia; 6 King Abdullah International Medical Research Center, Ministry of National Guard Health Affairs, Riyadh, Saudi Arabia; 7 Ministry of Health, Kuwait city, Kuwait; 8 Advanced Education in General Dentistry, Ministry of National Guard, Riyadh, Saudi Arabia

## Abstract

**Background:**

The relationship between malocclusion and the oral health related quality of life (OHRQoL) of children needs to be explored further as existing literature presents conflicting evidence. This study aims to determine the association between malocclusion and OHRQoL of 11–14-year-old children.

**Methods:**

This cross-sectional study was conducted among 250 caregiver/child dyads seeking orthodontic consultation at a tertiary care hospital. The OHRQoL was assessed using child perception questionnaire for 11–14-year-old children (CPQ_11–14_) and the severity of malocclusion was assessed using the Dental Aesthetic Index (DAI). CPQ_11–14_ scores ranged from 0 to 64, with lower scores representing better quality of life. Analysis of variance (ANOVA) was used to assess differences between domain and total CPQ_11–14_ scores.

**Results:**

The mean CPQ_11–14_ score was 19.89 ± 9.8. Mean scores for the oral symptoms, functional limitations, emotional well-being, and social well-being domains were 5.26 ± 3.22, 3.67 ± 3.58, 3.98 ± 3.89 and 2.08 ± 2.98, respectively. Normal or slight malocclusion was seen in 37.6%, definite malocclusion was seen in 22.4%, severe malocclusion in 15.2% and handicapping malocclusion in 24.8% of the subjects. In comparisons by pairs, it was found that children with handicapping malocclusion had significantly (*p* < 0.05) higher scores for the social well-being domain as compared with children having normal/minor malocclusion, indicating a poorer quality of life.

**Conclusion:**

Handicapping malocclusion had a significant negative impact on the social well-being domain of OHRQoL among 11–14-year-old children in this population.

## Background

Malocclusion is a developmental condition where there is a deflection from the normal relation or alignment of the teeth to other teeth in the same arch and/or to the teeth in the opposing arch [1]. Malocclusion is one of the most common oral conditions, with a prevalence ranging from 20 to 100% [2–5]. A previous study from Saudi Arabia reported a malocclusion prevalence of about 68% [6]. Due to the high prevalence of malocclusion, the World Health Organization considers malocclusion to be a significant public health problem [7].

The oral health related quality of life (OHRQoL) is defined as a composition of self-report, specifically pertaining to oral health that captures the functional, social and psychological impacts of oral disease [8]. Malocclusion affects the function, appearance, social life and self-esteem of individuals, which constitute the different constructs of OHRQoL [9]. Since malocclusion can be perceived differently by different individuals, it is essential to understand the impact of malocclusion from the patients’ perspective. The use of subjective measures along with the professionally determined treatment need has been shown to be beneficial in orthodontic treatment planning [10].

Several instruments have been used to assess the OHRQoL of children, Oral Health Impact Profile-14 (OHIP-14) [11], Child Oral Health Impact Profile (COHIP) [12], Early Childhood Oral Health Impact Scale (ECOHIS) [13], Child Perception Questionnaire (CPQ) [14], to mention a few. Though there are similarities in the constructs that are being assessed by these instruments, there are also differences. Some instruments tend to focus on the severity, whereas, others tend to focus on the frequency of oral impacts on the OHRQoL. Child Perceptions Questionnaire (CPQ_11–14_) has been widely used to assess the quality of life of children in the age group of 11–14 years [15]. The long version [6, 16] and the short version [17] of CPQ_11–14_ has been cross-culturally validated in the Middle-Eastern population.

The Dental Aesthetic Index (DAI) is one of several indices used to measure malocclusion [18]. DAI has been widely used in orthodontic research due to its simplicity, reproducibility and validity. DAI has been adopted by the World Health Organization as a cross-cultural index to assess malocclusion in epidemiological studies [19] and it has been used in different populations without modifications [20, 21].

The relationship between malocclusion and the OHRQoL of children needs to be explored further as existing literature presents conflicting evidence, where some reports suggest a significant association [6, 22], while some others do not find an association [23–25]. Therefore, this study aims to determine the association between malocclusion and OHRQoL of 11–14-year-old children.

## Methods

### Ethical approval

This study received ethical approval from the Institutional Review Board (IRB) of King Abdullah International Medical Research Center (Study Number: SP17/353/R), and is reported according to the Strengthening the Reporting of observational Studies in Epidemiology (STROBE) guidelines for observational studies [26]. Written informed consent was obtained from those parents who agreed to participate in this study.

### Study design and study subjects

This was a cross-sectional study based on a convenient sample from the orthodontic department at King Abdulaziz Medical City, Riyadh, Saudi Arabia, between July to December, 2017. Based on a previous study from a similar population [6], the expected effect size was assumed to be 0.15, with a standard deviation of 0.45, it was estimated that 250 participants would be required to obtain a power of 80% with a 95% confidence interval (95% CI). All children who were undergoing or had undergone orthodontic treatment in the past were excluded from the study.

### Measures of OHRQoL and clinical oral examination

Sociodemographic information was collected using a structured questionnaire from the parents who consented to participate in this study. The children completed the CPQ_11–14_ in the dental clinic waiting room just prior to the dental examination. CPQ_11–14_ consists of 4 close-ended questions for each of the four domains (oral symptoms, functional limitations, emotional well-being and social well-being). Each question was scored as: 0-never, 1-once\twice, 2-sometimes, 3-often and 4-every day\almost every day. The four domain scores were seperately added to give the total CPQ_11–14_ score, which ranged from 0 to 64. Lower scores represent better quality of life.

Malocclusion was assessed using Dental Aesthetic Index (DAI) (the number of variables = 10) [27]. The DAI measures 10 prominent traits of malocclusion, weighted on the basis of their relative importance, to produce a single score [1]. The 10 traits are: missing teeth, crowding, spacing, diastema, maxillary and mandibular anterior irregularity, overjet, reverse overjet, open bite and molar relationship. A DAI score of ≤ 25 indicates normal or minor malocclusion (no treatment needed); a score of 26–30 indicates moderate or definite malocclusion (treatment is elective); a score of 31–35 denotes severe malocclusion (treatment considered as highly desirable); and a score of ≥ 36 represents very severe (handicapping) malocclusion (mandatory treatment indicated) [27].

Five examiners underwent training and calibration for recording DAI at King Abdulaziz Medical City, Riyadh, Saudi Arabia. Kappa scores for inter- and intra-examiner reliability were > 0.80.

### Statistical analysis

Analysis of variance (ANOVA) or Student’s t-test was used to assess the differences between CPQ_11–14_ scores and socio-demographic variables. Multivariate analysis of variance (MANOVA) was used to assess the relationship between DAI and CPQ_11–14_ scores, controlling for the socio-demographic variables. Post hoc comparisons between pairs of malocclusion groups were conducted using Bonferroni test. A *p* value of < 0.05 was chosen as the cut-off for statistical significance. Data analysis was performed with the Statistical Package for Social Sciences (SPSS Inc., Chicago, IL, USA).

## Result

A total of 250 child-parent dyads participated in this study. The distribution of socio-demographic variables by CPQ_11–14_ mean scores is shown in Table 1. The domain and total scores of CPQ_11–14_ is shown in Table 2. The mean CPQ_11–14_ score was 19.89 ± 9.8. Table 3 presents severity of malocclusion across the different domain-specific scores of CPQ_11–14_. Mean scores for the oral symptoms, functional limitations, emotional well-being, and social well-being domains were 5.26 ± 3.22, 3.67 ± 3.58, 3.98 ± 3.89 and 2.08 ± 2.98, respectively. Domain-specific scores showed that the highest mean score was observed for the oral symptoms domain and the lowest score was observed for the social well-being domain. Normal or slight malocclusion was seen in 37.6%, definite malocclusion was seen in 22.4%, severe malocclusion in 15.2% and handicapping malocclusion in 24.8% of the subjects. Children with handicapping malocclusion had significantly higher scores (indicating a poorer quality of life) for the social well-being domain as compared with children who had normal/minor malocclusion. There was no statistically significant difference in domain or total CPQ_11–14_ scores by any of the sociodemographic variables.

**Table 1:** Total Scores on the Child Perception Questionnaire (CPQ_11–14_) for the socio-demographic variables

Variables		N (%)	CPQ_11–14_ Mean (SD)	*p* value^b^
Age	11 years	106 (42.4)	20.14 (9.90)	0.20
	12 years	55 (22.0)	18.65 (9.01)	
	13 years	49 (19.6)	22.04 (10.75)	
	14 years	40 (16.0)	18.02 (9.26)	
Gender	Male	64 (25.6)	19.45 (10.88)	0.68
	Female	186 (74.4)	19.97 (9.44)	
Mother’s educational level^a^	Less than high school	84 (33.6)	20.68 (10.16)	0.49
	High school or diploma	73 (29.2)	19.57 (8.43)	
	Bachelor	77 (30.8)	19.86 (9.39)	
	Master and above	11 (3.2)	18.75 (21.78)	
Father’s educational level^a^	Less than high school	47 (18.8)	20.91 (11.09)	0.26
	High school or diploma	97 (38.8)	20.11 (9.10)	
	Bachelor	83 (33.2)	18.79 (8.85)	
	Master and above	17 (5.2)	24.00 (17.45)	
Family monthly income^a^	Less than 5000 SAR	42 (16.8)	22.09 (12.36)	0.40
	5000–15,000 SAR	88 (35.2)	20.10 (9.02)	
	15,000–20,000 SAR	72 (28.8)	18.94 (8.12)	
	More than 20,000 SAR	38 (15.2)	19.44 (11.89)	

^a^Presence of missing values

^b^ANOVA or Student’s t-test

*SAR* Saudi Arabian Riyals

**Table 2:** Domain and Total Scores on the Child Perception Questionnaire (CPQ_11–14_) for 11- to 14-year-old children in the sample

Items	No. of Items	Possible Range	Observed Range	Mean (SD)
Oral symptoms	4	0–16	0–16	5.26 (3.22)
Functional limitation	4	0–16	0–16	3.67 (3.58)
Emotional well-being	4	0–16	0–16	3.98 (3.89)
Social well-being	4	0–16	0–16	2.08 (2.98)
Total CPQ_11-14_ score	16	0–64	2–62	19.89 (9.80)

**Table 3:** Domain and Total Scores on the Child Perception Questionnaire for 11- to 14-year-old Children (CPQ_11–14_) by Dental Aesthetic Index (DAI) Malocclusion Severity

Severity of Malocclusion	N (%)	Oral health related quality of life (OHRQoL)				
		**Oral symptoms** **Mean(SD)**	**Functional limitation** **Mean(SD)**	**Emotional well-being** **Mean(SD)**	**Social well-being** **Mean(SD)**	**Total CPQ**_**11-14**_** score** **Mean(SD)**
Normal/Minor	94 (37.6)	5.28 3.17)	3.55 (3.60)	3.92 (3.97)	1.51 (2.33)^a^	19.62 (9.83)
Definite	56 (22.4)	4.80 (2.85)	3.53 (3.25)	3.43 (3.54)	1.75 (2.54)	18.59 (8.14)
Severe	38 (15.2)	5.71 (3.86)	3.57 (4.38)	3.68 (4.26)	2.65 (4.08)	20.42 (13.05)
Very severe	62 (24.8)	5.40 (3.22)	3.87 (3.38)	4.80 (3.76)	2.88 (3.20)^a^	21.16 (8.83)
Total Mean Score	250 (100.0)	5.26 (3.22)	3.67 (3.58)	3.98 (3.89)	2.08 (2.98)	19.89 (9.80)

^a^Superscripts indicate which pairs of malocclusion severity groups were statistically different (*p* < 0.05) using Multivariate analysis of variance

## Discussion

Subjective perception towards oral health is an important measure as it directly influences the oral health behaviors. In this study, handicapping malocclusion was found to negatively impact the social well-being domain of CPQ_11–14_. Social well-being domain reflects the child’s quality of life with regard to the appearance of their dentition, self-esteem related to oral health, and their interaction with peers [28]. Similar associations between DAI scores and social well-being domain were reported in other studies as well [6, 22]. DAI is an orthodontic treatment index that is designed to assess the orthodontic needs based on the socially defined esthetic standards [29]. Therefore, it is recognized that DAI is sensitive to the social and emotional domains of quality of life measures.

In this study, children with definite malocclusion had the lowest CPQ_11–14_ scores and children with handicapping malocclusion had the highest CPQ_11–14_ scores. A clear gradient for the total CPQ_11–14_ scores was not observed across the four categories and the difference between groups were not statistically significant. Dawoodbhoy et al. [6] and Locker et al. [30] also reported a lack of a clear gradient across different levels of malocclusion. However, Kassis et al.[31] observed a clear ascending gradient for CPQ_11–14_ score across ascending categories of orthodontic treatment need. The difference may be due to differences in the sample characteristics and the distribution of subjects and sample sizes across the four treatment need categories.

As with any quality of life study, respondents’ socio economic factors are considered to be important confounders. Foster Page et al. [32] found that the mean emotional well-being domain score was higher for girls than for boys. However, in this study, no significant difference in the total CPQ_11–14_ scores were observed between the genders. Other studies have also reported that females and males are equally affected on all the four domains of CPQ_11–14_ [33, 34].

The OHRQoL measures have the potential to provide an unique perspective of how oral conditions affect an individual’s everyday life, which is crucial for the dentist to understand the patients’ treatment needs [24]. Our findings suggest that clinicians should consider subjective measures like the quality of life along with their clinical assessment during treatment planning. These subjective measures can complement the traditional or the professionally determined orthodontic treatment needs.

The results of this study should be interpreted with caution due to limitations relating to the lack of representativeness of the study sample. This was a hospital-based study which recruited a convenient sample from patients reporting to the orthodontic department at the dental centre. This limits the ability to extrapolate the study findings to the general population. In addition, CPQ_11–14_ is not a malocclusion specific OHRQoL measure, and as such, it captures the effects on the quality of life attributed by the general oral health conditions.

## Conclusions

Handicapping malocclusion had a significant negative impact on the social well-being domain of OHRQoL among 11–14-year-old children in this population. The findings of this study provides additional evidence that handicapping malocclusion negatively impacts the oral health related quality of life.

## Data Availability

Data used in this study is available from the corresponding author upon reasonable request.

## Abbreviations

CPQ: Child perception questionnaire
DAI: Dental Aesthetic Index
OHRQoL: Oral health related quality of life
